# Li-Hong Tang alleviates dextran sodium sulfate-induced colitis by regulating NRF2/HO-1 signaling pathway and gut microbiota

**DOI:** 10.3389/fphar.2024.1413666

**Published:** 2024-05-30

**Authors:** Hong Gu, Yuwen Tian, Jingjing Xia, Xiaoyue Deng, Jian Chen, Tunyu Jian, Jiong Ma

**Affiliations:** ^1^ Jiangyin Hospital Affiliated to Nanjing University of Chinese Medicine, Jiangyin, China; ^2^ Jiangsu Key Laboratory for the Research and Utilization of Plant Resources, Institute of Botany, Jiangsu Province and Chinese Academy of Sciences, Nanjing, China

**Keywords:** Li-Hong Tang, ulcerative colitis, intestinal inflammatory disease, Chinese medicine, botanical drug, gut microbiota

## Abstract

**Introduction:**

Ulcerative colitis (UC) is marked by recurring inflammation. Existing treatments are ineffective and may have toxic side effects. Thus, new therapeutic agents are urgently needed. We studied the botanical formula “Li-Hong Tang (LHT)", which contains two main ingredients, *Salvia plebeia* R. Br and *Rhodiola crenulata* (Hook. f. et Thoms.) H. Ohba. In this study, we aimed to identify the effects of LHT on UC and explore its potential mechanism.

**Methods:**

LHT was analyzed using a mass spectrometer (MS). DSS at a dose of 2.5% was utilized to develop UC in mice. The administered groups received low, medium, and high dosages (0.32 g/kg, 0.64 g/kg, and 1.28 g/kg) of LHT and the positive medication, sulfasalazine (0.2 g/kg), respectively. Body weight, disease activity index (DAI) score, colon length, spleen index, serum myeloperoxidase (MPO), nitric oxide (NO), superoxide dismutase (SOD) and inflammatory factor concentrations were monitored. The expression of NRF2 and HO-1 in colonic tissues was evaluated by immunohistochemistry. 16S rDNA sequencing was employed to investigate alterations in the gut microbiota of the mice, aiming to elucidate the extent of LHT’s impact.

**Results:**

LHT may ameliorate DSS-induced colitis in mice by lowering inflammation, reducing oxidative stress, restoring the intestinal barrier, and influencing the NRF2/HO-1 pathway. Moreover, LHT treatment exhibited a regulatory effect on the gut microbiota, characterized by elevated levels of Patescibacteria, Verrucomicrobiota, *Candidatus_Saccharimonas*, *Lactobacillus*, and *Ligilactobacillus* levels while decreasing *Oscillibacter* and *Colidextribacte*r levels. Further study indicated that MPO, NO, and inflammatory factors were positively correlated with *Oscillibacter*, *Colidextribacter*, *Escherichia-Shigella*, *Anaerostines*, and negatively with *Lactobacillus*, *Clostridiales_unclassified*, *Candidatus_Saccharimonas,* and Patescibacteria. Furthermore, colony network analysis revealed that *Lactobacillus* was negatively associated with *Oscillibacter* and *Colidextribacter*, whereas *Oscillibacter* was positively related to *Colidextribacter*.

**Conclusion:**

LHT protects against DSS-induced mice by inhibiting the inflammatory response, oxidative stress, and mucosal injury. The protective role may involve regulating the NRF2/HO-1 signaling pathway and gut microbiota.

## 1 Introduction

Ulcerative colitis (UC), a chronic intestinal inflammatory disease (IBD) characterized by degeneration, loss of crypt structure, and infiltration of inflammatory cells in the intestinal epithelium, primarily affects the mucosa of the colon (Fei and Xu, 2016). Its recurrent nature and long duration of the condition harm people’s quality of life and increase the economic burden (Lindsay et al., 2015). With the advancement of modern medicine, there has been an increasing knowledge of UC pathophysiology, yet many intricate elements remain unresolved. Environmental variables, genetic vulnerability, intestinal epithelial barrier disturbance, and immune response problems are all thought to have a role in the development of UC (Huang et al., 2022). It is becoming increasingly clear that disturbance of the gut microbiota plays a critical role in the development of UC.

Within Traditional Chinese medicine (TCM) theory, ulcerative colitis (UC) is diagnosed based on symptoms including dysentery, diarrhea, intestinal wind, and blood in the stool. Internal dampness-heat and spleen and kidney weakening are crucial contributors to the disease’s development (Wu et al., 2022). Treatment for UC focuses on strengthening the spleen and kidney functions while eliminating dampness and heat. This approach is believed to alleviate symptoms and provide benefits for patients with UC. With its principles of precision therapy and dialectic medication, TCM may reduce oxidative stress, suppress the inflammatory response, control gut microbiota, and repair the intestinal mucosal barrier, thereby potentially treating UC (Zhou et al., 2023).

*Pulsatilla* Decoction (Bai Tou Weng Tang) is a formula used in TCM to treat UC (Li et al., 2020; Deng et al., 2022). Li-Hong Tang (LHT) is a refined prescription formula derived from the ancient Chinese medical prescription “Bai Tou Weng Tang” but has undergone modifications to enhance its therapeutic efficacy and improve patient outcomes. The ingredients of LHT include seven botanical drugs: *Salvia plebeia* R. Br., *Rhodiola crenulata* (Hook. f. et Thoms.) H. Ohba, *Lithospermum erythrorhizon* Siebold & Zucc., *Rhus chinensis* Mill., *Pulsatilla chinensis* (Bunge) Regel, *Codonopsis pilosula* (Franch.) Nannf., and *Atractylodes macrocephala* Koidz. The combination of these botanical drugs clears heat, cools the blood, detoxifies, disperses bruises, and benefits vital energy and intestines. Among them, *S. plebeia* R. Br and *R. crenulata* (Hook. f. et Thoms.) H. Ohba are the main ingredients. A flavonoid metabolite of Salvia plebeia R. Br. has anti-tumor properties by blocking the PI3-K/Akt pathway (Jiang et al., 2017). *Rhodiola crenulata* (Hook. f. et Thoms.) H. Ohba is well-known for its anti-inflammatory, antioxidant, antibacterial, and anti-tumor properties (Bai et al., 2019). A recent study found *R. crenulata* (Hook. f. et Thoms.) H. Ohba can alleviate dextran sulfate sodium (DSS)-induced colitis through a mechanism involving the modification of the gut microbiota (Liu et al., 2023). It is worth noting that disruptions of the gut microbiota are a significant hallmark of UC, and several studies have shown that fecal microbiota transplantation can alleviate UC symptoms (He et al., 2019; Wu et al., 2019). It is unknown whether LHT affects gut microbiota. This study examined the modulation of gut microbiota structure by LHT using a DSS-induced mouse model. This study deeply explored LHT’s mechanism of action for treating UC, focusing on its ability to reduce inflammatory responses and oxidative stress and alter gut microbiota composition. This work lays the groundwork for clinical trials of LHT for UC. It also proposes a TCM treatment plan with a clear mechanism and potentially precise efficacy, offering significant therapeutic value.

## 2 Materials and methods

### 2.1 Reagents

DSS was purchased from MP Biomedicals (Solon, OH, United States) (Batch No.: 160110); sulfasalazine (SASP) (Batch No.: S838221) was purchased from Shanghai McLean Biochemical Technology Co. TNF-α (Batch No.: EK282), IL-1β (Batch No.: EK201B), IL-6 (Batch No.: EK206), and IL-18 (Batch No.: EK218) ELISA kits were purchased from Hangzhou Lianke Bio-technology Co. Ltd. Crypto Blood Kit (Batch No.: C027-1-1) and myeloperoxidase (MPO) (Batch No.: A044-1-1) were purchased from Nanjing Jiancheng Bioengineering Institute; nitric oxide (NO), superoxide dismutase (SOD) kits were purchased from Shanghai Biyuntian Biotechnology Company (Batch No.: S0021S, S0101M). Antibody NRF2 and HO-1 were purchased from Proteintech (Batch No.: 16396-1-AP, 10701-1-AP).

### 2.2 Preparation of LHT extractions

LHT was purchased from Jiangyin Hospital of Traditional Chinese Medicine (Wuxi, China), and the seven botanical drugs of LHT and their dosage are shown in Table 1. LHT was extracted using the traditional aqueous decoction method, in which the botanicals were extracted with three times the volume (w/v) of boiling water heated for 1 h at 100°C. The filtrates were combined after two extractions, and then the extracts were distilled into an extract under reduced pressure and stored at 4°C.

**TABLE 1:** Ingredients of LHT.

Chinese name	Latin name	Dosage	Part of the durg used
Lizhicao	*Salvia plebeia* R.Br.	30g	Whole plant
Hongjingtian	*Rhodiola crenulata* (Hook.f.et Thoms) H. Ohba	30g	Rhizome
Zicao	*Lithospermum erythrorhizon* siebold & Zucc	15g	Root
Wubeizi	*Rhus chinensis* Mill	5g	Inset galla
Baitouweng	*Pulsatilla chinensis* (Bunge)Regel	10g	Root
Dangshen	*Codonophsis pilosula* (Franch.)Nannf.	15g	Root
Baizhu	*Atractylodes macrocephala* Koidz	15g	Rhizome

The composition of LHT was analyzed as follows: the samples were separated using a YMC-Pack ODS-AQ column (4.6 mm i.d., 250 mm i.d., 5 μm, Japan). Gradient elution was performed using mobile phase water (solvent A) and acetonitrile (solvent B). In terms of solvent B, the gradient program was set as follows: from 10% to 100% at 0–60 min. The mobile phase flow rate was set to 1.0 mL/min. However, a post-column split reduced the flow rate entering the MS analysis system to 0.5 mL/min. The column temperature was maintained at 30°C. Detection was performed using a diode array detector set at 254 nm.

MS detection was performed using an ESI interface on an Agilent 6530 Q-TOF MS machine. The capillary voltage was 4000 V for positive ion detection mode and 3500 V for negative ion detection mode, respectively. Other parameters were set as follows: drying gas (N_2_) flow rate of 5.0 L/min, drying gas temperature of 300°C, nebulizer pressure of 45 psig, sheath gas temperature of 400°C, and sheath gas flow rate of 12 L/min. Accurate measurement of all mass peaks yielded a mass range of 100–1,00 m/z. Agilent Mass Hunter Acquisition software (Agilent Technologies, CA, United States) handled the operations, acquisition, and data analysis.

### 2.3 Animals

C57BL/6J mice were used to create the colitis model; they were kept at 24–26 g. With License No. SCXK (Su) 202-0-0009 mice were acquired from Shanghai Sino-British SIPPR/BK Laboratory Animal Co. for the experiment; the mice were housed in conventional settings with a temperature of 20°C–24°C and a humidity of 40%–60%. They were also fed and acclimated for 7 days. The Animal Care and Use Ethics Committee of the Nanjing University of Chinese Medicine (Nanjing, China) approved these studies.

After 7 days of acclimation feeding, a total of 36 mice were randomly divided into six groups (6 mice in each group): control (Con), model (UC), LHT low dose (LHT-L), LHT middle dose (LHT-M), LHT high dose (LHT-H), and positive drug SASP. Except for the control group, mice in the other groups were given free access to drinking water containing DSS at a concentration of 2.5% for 7 days. Two days after drinking DSS water, the LHT-L, LHT-M, and LHT-H groups received daily gavage doses of 0.32 g/kg, 0.64 g/kg, and 1.28 g/kg LHT extraction, respectively. Notably, the LHT-M dose corresponds to the clinical dose used in humans. The SASP group received 0.2 g/kg of SASP, whereas the normal and DSS groups received the same amount of water. The mice were administered continuously for 5 days. Samples from the colon and spleen were collected to determine colon length and spleen index.

### 2.4 Disease activity index (DAI) score

On the last day of the experiment, DAI scores were administered on the following scale.

**Table:** 

	Description	Score
Degree of weight reduction	normal	0
	1%–5%	1
	5%–10%	2
	10%–20%	3
	>20%	4
Hardness or softness of stool	normal	0
	soft stool	1
	meager stool	2
	diarrhea	3
	severe diarrhea	4
Degree of fecal occult blood	normal	0
	occult blood	1
	weak positive	2
	positive	3
	strong positive	4

### 2.5 H&E staining

Colon tissues were rinsed with PBS buffer, and an adequate size was removed and fixed with 4% paraformaldehyde for 24 h. After fixation, tissues were dried, embedded in paraffin wax, sectioned to a thickness of 5 μm, stained with H&E staining, and examined under a light microscope.

### 2.6 Biochemical analysis

After standing for 0.5 h, blood was collected from the mice. After 30 min of standing at room temperature, the supernatant was isolated by centrifugation at 4°C, 3,000 g for 15 min. The supernatant was collected. MPO, NO, and SOD were analyzed in accordance with commercial kit guidelines in the plasma. TNF-α, IL-6, IL-1β, and IL-18 levels in serum were measured using ELISA.

### 2.7 Detection of NRF2 and HO-1 expression in colonic tissues by immunohistochemistry

Colon sections were dewaxed to water for immunohistochemistry to detect NRF2 and HO-1 expression. The slides were incubated with anti-NRF2 and anti-HO-1 at 4°C overnight, followed by the corresponding secondary antibodies. Sections were stained with diaminobenzidine, re-stained with hematoxylin staining solution, and examined under a light microscope.

### 2.8 16S-rDNA sequencing

At the end of the experiment, the intestinal contents of the mice were collected in EP tubes. Total genomic DNA was isolated from mice’s fecal samples using a fecal DNA kit. Agarose gel electrophoresis was used to assess the purity of the DNA extraction, and UV spectrophotometry was used to quantify the DNA. The NovaSeq 6,000 was used to sequence and analyze the data. Sequencing results were provided by Hangzhou Lianchuan Biotechnology Co.

### 2.9 Statistical processing

The experiment results were reported as mean ± standard error of the mean (SEM). In immunohistochemistry, Fisher’s least significant difference was utilized. The remaining variables were analyzed using one-way ANOVA and Dunnett’s test. *p* < 0.05 was regarded as statistically significant. The GraphPad Prism 9.1.1 software (GraphPad Prism, San Diego, California) was used for data processing and graphing.

## 3 Results

### 3.1 Analysis of the composition of LHT

**FIGURE 1:**
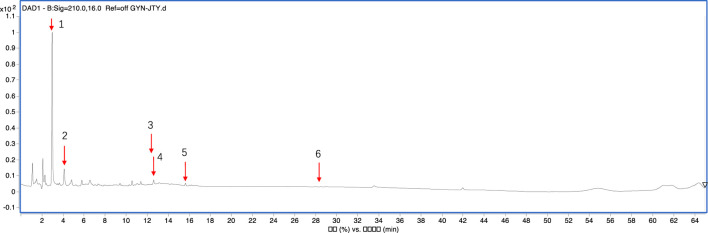
Total ion chromatograms of the primary metabolites in the LHT.

The mass spectrometer was used to identify LHT components (Figure 1), and six metabolites were identified. Table 2 shows the compositions, retention time, and identification in LHT.

**TABLE 2:** Compositional identification of LHT.

No.	Compositions	Retntion time(min)	Identification	Formula	M.W.
1	*Rhus chinensis* Mill.	2.99	Gallic acid	C_7_H_6_O_5_	170.02
2	*Rhodiola crenulata* (Hook.f. et Thoms.) H.Ohba	4.17	Salidroside	C_14_H_20_O_7_	300.12
3	*Codonopsis pilosula*(Franch.) Nannf.	12.42	Lobetyolin	C_20_H_28_O_8_	396.18
4	*Salvia plebeia* R. Br	12.66	Homoplantanginin	C_22_H_48_O_4_	462.12
5	*Pulsatilla Chinensis* (Bunge) Regel	15.72	Hydroxybetulinic acid or Hederagenin	C_30_H_48_O_4_	472.12
6	*Atractylodes macrocephala* Koidz.	28.57	Atractylenolides III	C_15_H_20_O_3_	248.14

### 3.2 LHT treatment reduces mice symptoms with DSS-Induced colitis

**FIGURE 2:**
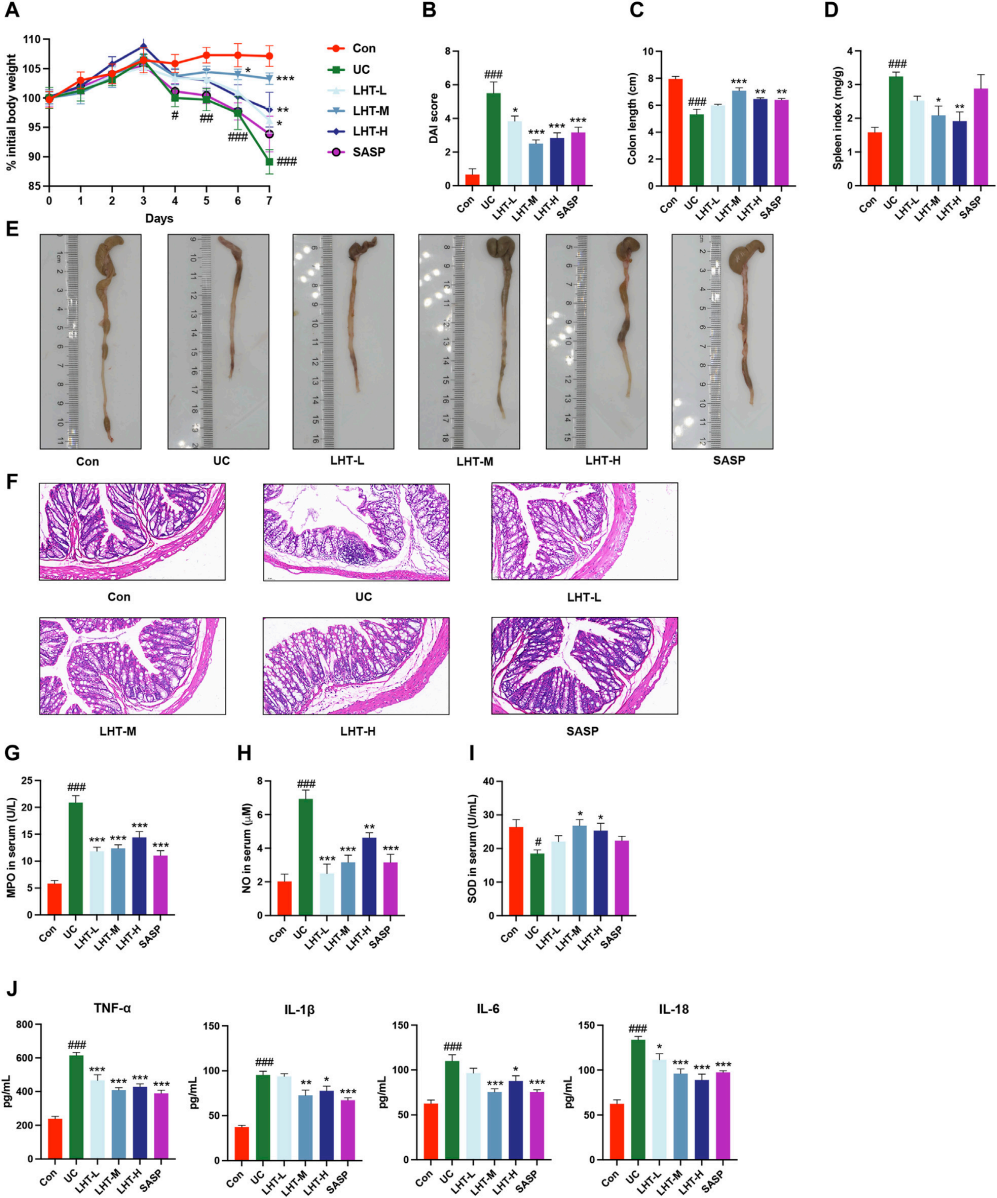
Alleviation of DSS-induced colitis in mice by LHT treatment. **(A)** Comparison of daily body weights of mice. **(B)** DAI scores of mice on day 7. **(C)** Mouse colon length. **(D)** Mouse spleen index. **(E)** Representative pictures of the colon. **(F)** H&E staining of colon tissue sections (scale bar of 50 μm in the figure). Serum concentrations of **(G)** MPO, **(H)** NO, **(I)** SOD, and **(J)** inflammatory factors (TNF-α, IL-1β, IL-6, IL-18) in mice. The results were expressed as mean ± SEM, n = 6/each group, ^#^ *p* < 0.05, ^##^ *p* < 0.01 and ^###^ *p* < 0.001, compared with Con group, ^*^ *p* < 0.05, ^**^ *p* < 0.01 and ^***^ *p* < 0.001, compared with UC group.

We explored the weight, DAI score, colon length, and biochemical standard to thoroughly evaluate the therapeutic benefits of LHT on colonic inflammation. DSS consumption resulted in varying degrees of weight loss compared to starting body weight. On day 4, the UC group showed significance (*p* < 0.05). Compared to the model group, all LHT treatments provided relief from weight loss by the final day (*p* < 0.05, Figure 2A). LHT also restored DSS-induced DAI reduction, colon length shortening, and splenic index increase (Figures 2B–E). H&E staining confirmed the presence of intact colonic mucosa with well aligned and dispersed crypts, consistent with healthy tissue. However, the colonic mucosa was injured after DSS consumption, as demonstrated by crypt distortion and inflammatory infiltration (Figure 2F). Surprisingly, the LHT administration group showed various degrees of improvement. Furthermore, LHT and SASP therapy also reduced DSS-induced increases in MPO, NO, TNF-α, IL-1β, IL-6, and IL-18, while elevating SOD levels (*p* < 0.05, Figures 2G–J).

### 3.3 LHT modulates the NRF2/HO-1 signaling pathway

**FIGURE 3:**
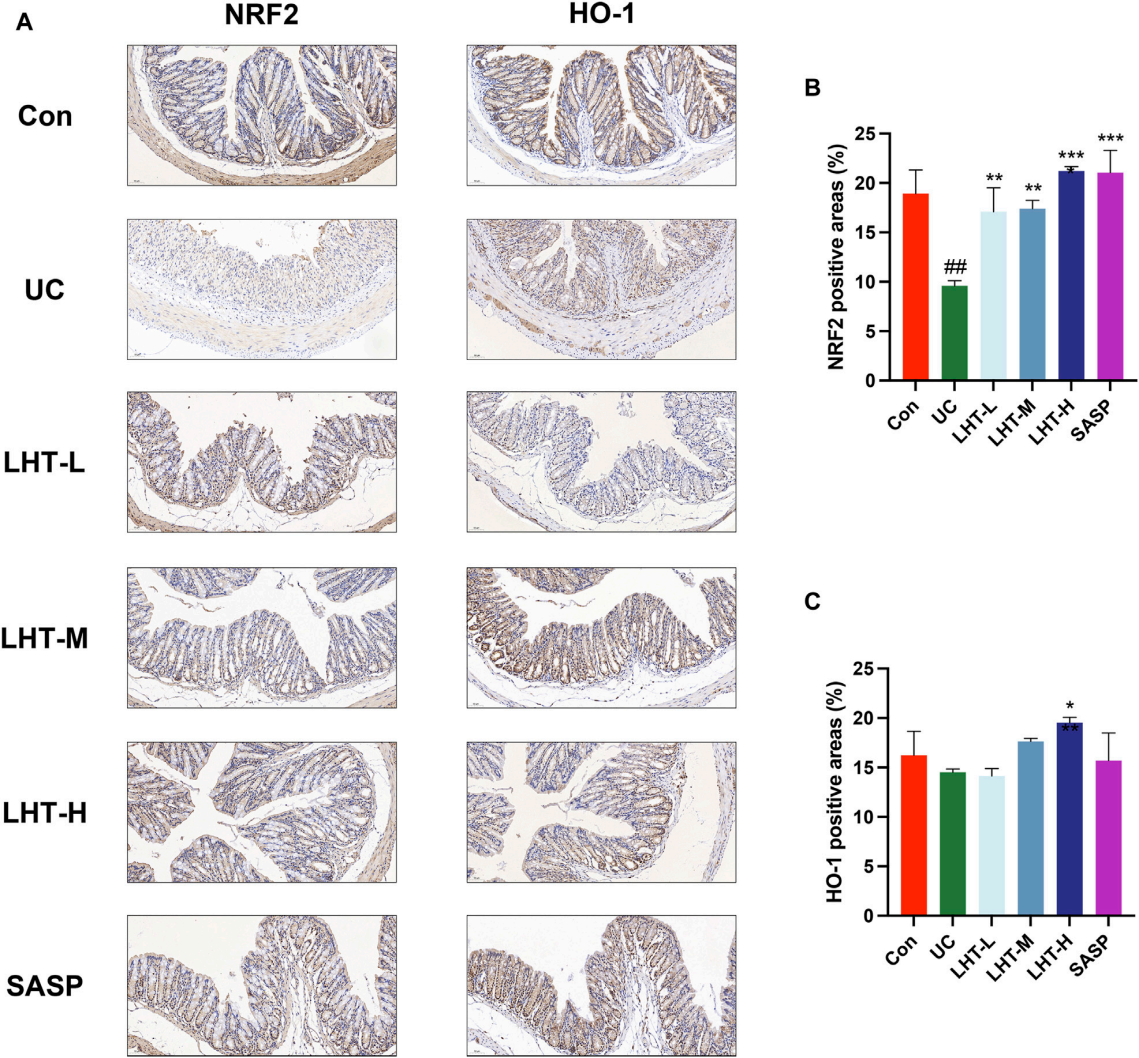
Modulation of the NRF2/HO-1 signaling pathway by LHT. **(A)** Immunohistochemical staining of NRF2 and HO-1 in colon tissue (scale bar of 50 μm in the figure). **(B)** Analysis of the positive area levels of NRF2. **(C)** Analysis of the positive area levels of HO-1. The results were expressed as mean ± SEM, n = 3/each group, ^#^ *p* < 0.05, ^##^ *p* < 0.01 and ^###^ *p* < 0.001, compared with Con group, ^*^ *p* < 0.05, ^**^ *p* < 0.01 and ^***^ *p* < 0.001, compared with UC group.

Immunohistochemical results showed that compared with the normal group, the positive area level of NRF2 in the colonic tissues of mice with IBD was significantly decreased (*p* < 0.01) and increased significantly in the LHT-L, LHT-M, LHT-H, and SASP groups (*p* < 0.01). The positive area level of HO-1 in the colonic tissues of mice in the model group tended to decrease (*p* = 0.45) and increased significantly in the LHT-H group (*p* < 0.05) (Figure 3).

### 3.4 LHT treatment increases gut microbiota diversity

**FIGURE 4:**
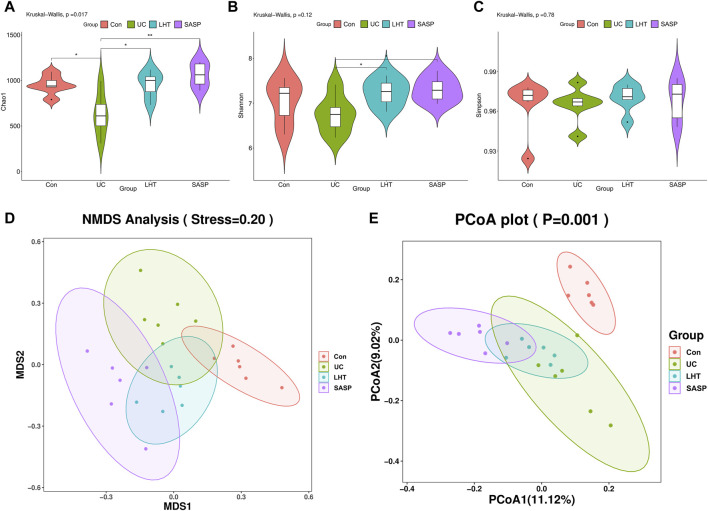
The diversity of the gut microbiota is improved by LHT treatment. **(A)** Chao1 level. **(B)** Shannon level. **(C)** Simpson level. **(D)** NMDS. **(E)** PCoA. n = 6/each group. The results were expressed as mean ± SEM, n = 6/each group, ^#^ *p* < 0.05, ^##^ *p* < 0.01 and ^###^ *p* < 0.001, compared with Con group, ^*^ *p* < 0.05, ^**^ *p* < 0.01 and ^***^ *p* < 0.001, compared with UC group.

In the next experiment, we focused on exploring the effects of the medium-dose group of LHT on the gut microbiota. The α-diversity was investigated using various metrics (Chao1, Shannon, Simpson), where Chao1 represents species richness, and Shannon and Simpson represent species diversity. The results showed that LHT improved the tendency of Chao1 and Shannon to decrease (*p* < 0.05) but not Simpson (Figures 4A–C). The NMDS and PCoA results revealed a significant difference in colonial similarity between the Con and UC groups (Figures 4D,E). However, the colony structure was altered following LHT therapy. In short, these results implied that LHT had the potential to increase the richness and variety of gut microbiota.

### 3.5 LHT treatment reverses disruption of the gut microbiota caused by DSS

**FIGURE 5:**
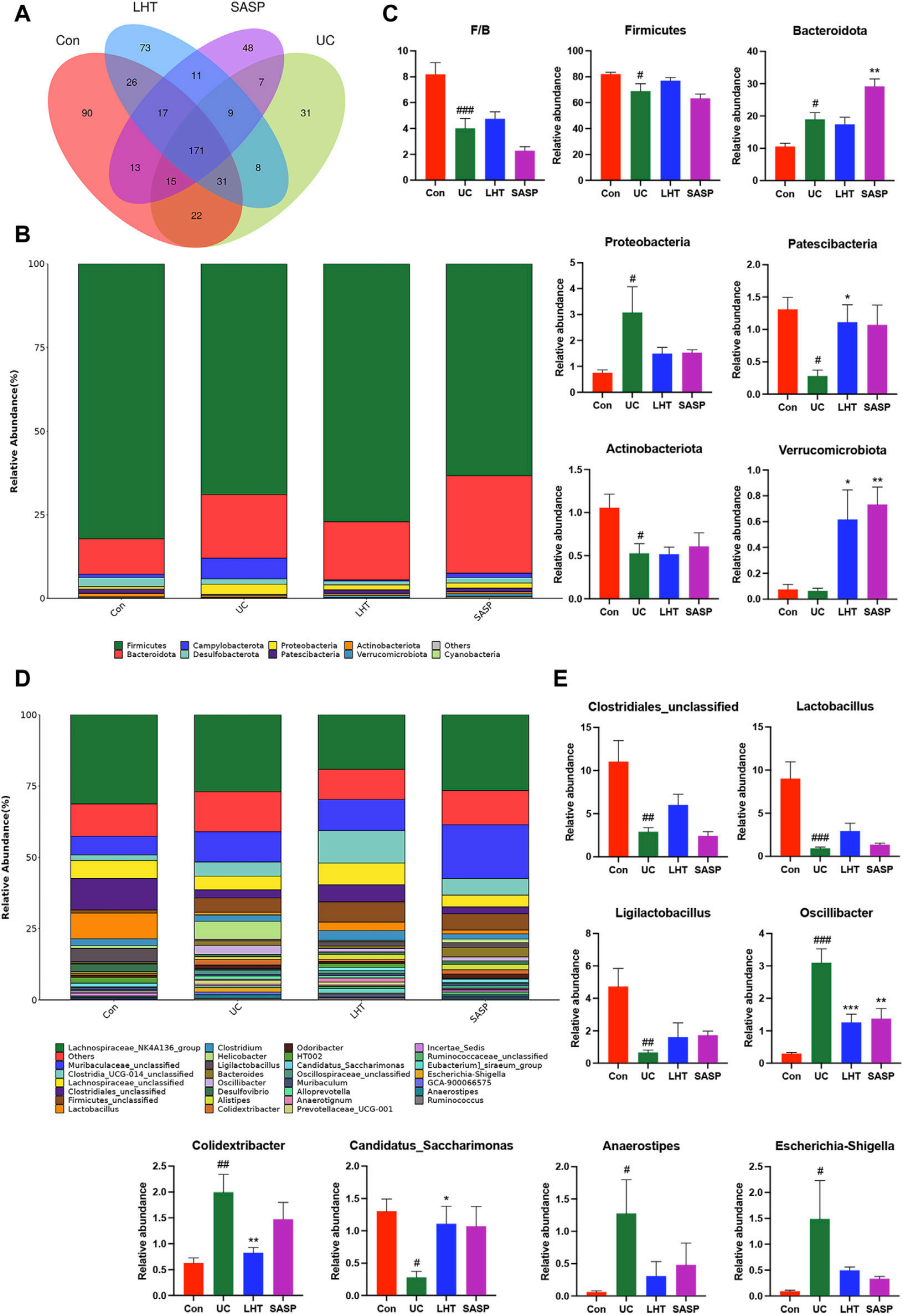
DSS-induced gut microbiota disruption was ameliorated by LHT treatment. **(A)** Venn diagram for multiple group comparisons. **(B)** Compositional characteristics at the phylum level. **(C)** Relative abundance of representative bacteria at the phylum level. **(D)** Characteristics of genus-level composition. **(E)** Relative abundance of representative bacteria at the genus level. Results are presented as mean ± SEM, n = 6/each group, ^#^ *p* < 0.05, ^##^ *p* < 0.01 and ^###^ *p* < 0.001, compared with Con group, ^*^ *p* < 0.05, ^**^ *p* < 0.01 and ^***^ *p* < 0.001, compared with UC group.

The Venn diagram revealed that the Con, UC, LHT, and SASP groups shared 171 overlapping ASVs and that the ASVs unique to the UC group dropped following DSS treatment. In contrast, the ASVs unique to the UC group increased after LHT and SASP administration (Figure 5A). We then concentrated on the specific alterations in the abundance of bacterial microbiota at the phylum and genus levels. DSS ingestion led to a considerable enrichment of Bacteroidota and Proteobacteria, as well as a significant decrease (*p* < 0.05) in the relative abundance of Firmicutes, Patescibacteria, and Actinobacteriota (Figures 5B,C). Following LHT treatment, the relative abundance of Patescibacteria and Verrucomicrobiota increased significantly (*p* < 0.05), but the relative abundance of Proteobacteria dropped (*p* = 0.31). It also had a high relative abundance of Firmicutes (*p* = 0.28).

*Clostridiales_unclassified*, *Lactobacillus*, *Ligilactobacillus*, and *Candidatus_Saccharimonas* had lower levels in the UC group (*p* < 0.05), whereas *Oscillibacter*, *Colidextribacter*, *Anaerostipes*, *Escherichia-Shigella* levels were significantly higher (*p* < 0.05) (Figures 5D,E). The abundance of *Oscillibacter* and *Colidextribacter* was dramatically reduced following LHT treatment (*p* < 0.05). Our research indicated that LHT can modulate the gut microbiota composition, potentially alleviating DSS-induced structural issues.

### 3.6 LHT treatment for functional modulation of gut microbiota

**FIGURE 6:**
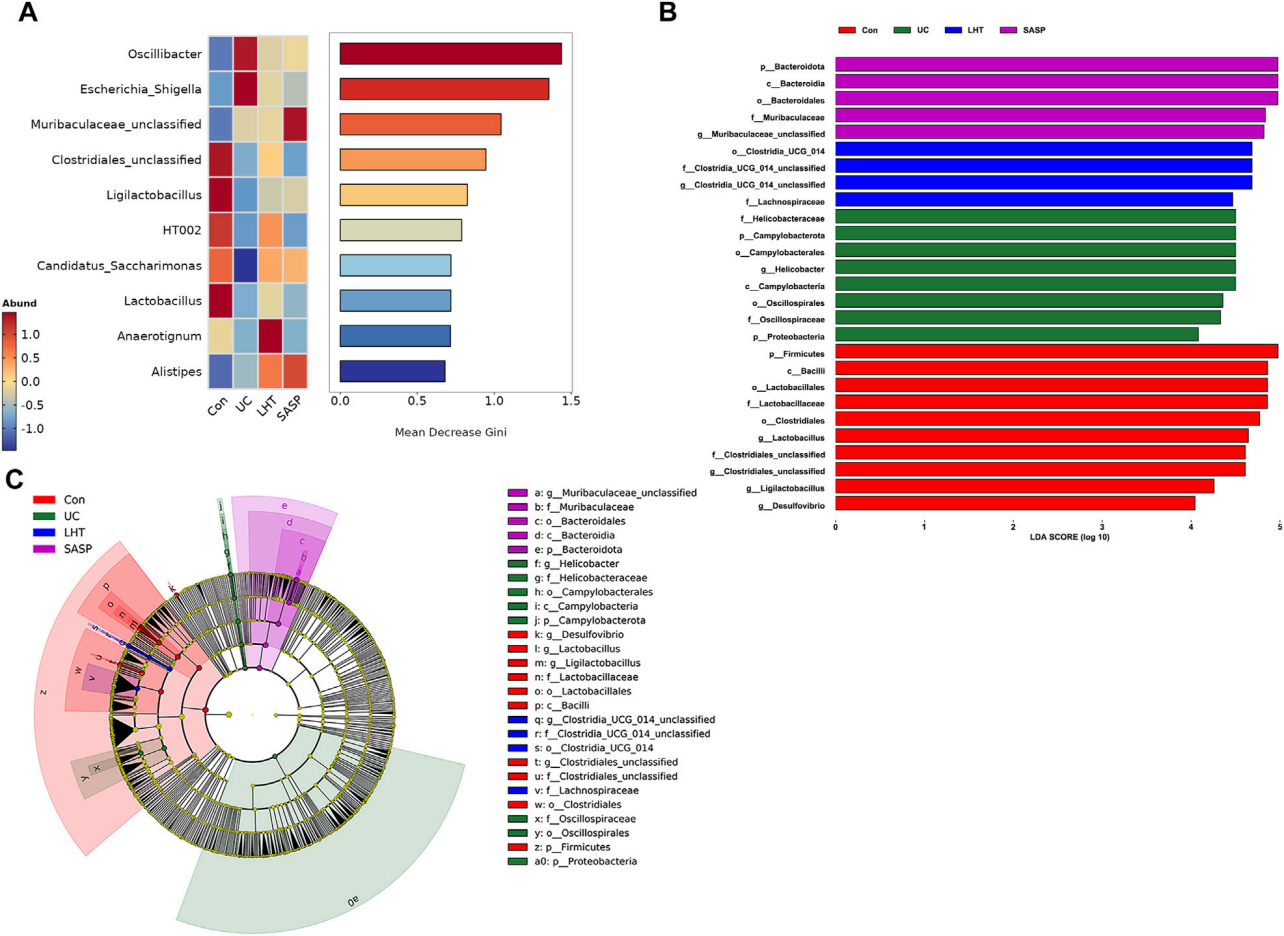
Remarkable enrichment and pathway prediction of core differential bacteria. **(A)** Genus-level random forest analysis. **(B, C)** For the LEfSe analysis, the LDA score was thresholded at 4.0.

We employed LEfSe and random forest analysis to examine gut microbial indicators further. The results revealed that the model group’s gut microbiota was characterized by an increase in the relative abundance of *Oscillibacter*, *Escherichia_Shigella*, and a decrease in the relative abundance of *Candiadatus_Saccharimonas* (Figure 6A). Specific taxa were found in each group’s gut microbiota, and when the LDA score was adjusted to 4.0, the normal group had a substantial enrichment of the genera *Lactobacillus*, *Clostridiales_unclassified*, and *Ligilactobacillus* compared to the model group (Figures 6B,C). LHT intervention treatment reversed this trend, with the enrichment of *Anaerotignum* and *Clostridia_UCG_014_unclassified* observed in the gut microbiota.

**FIGURE 7:**
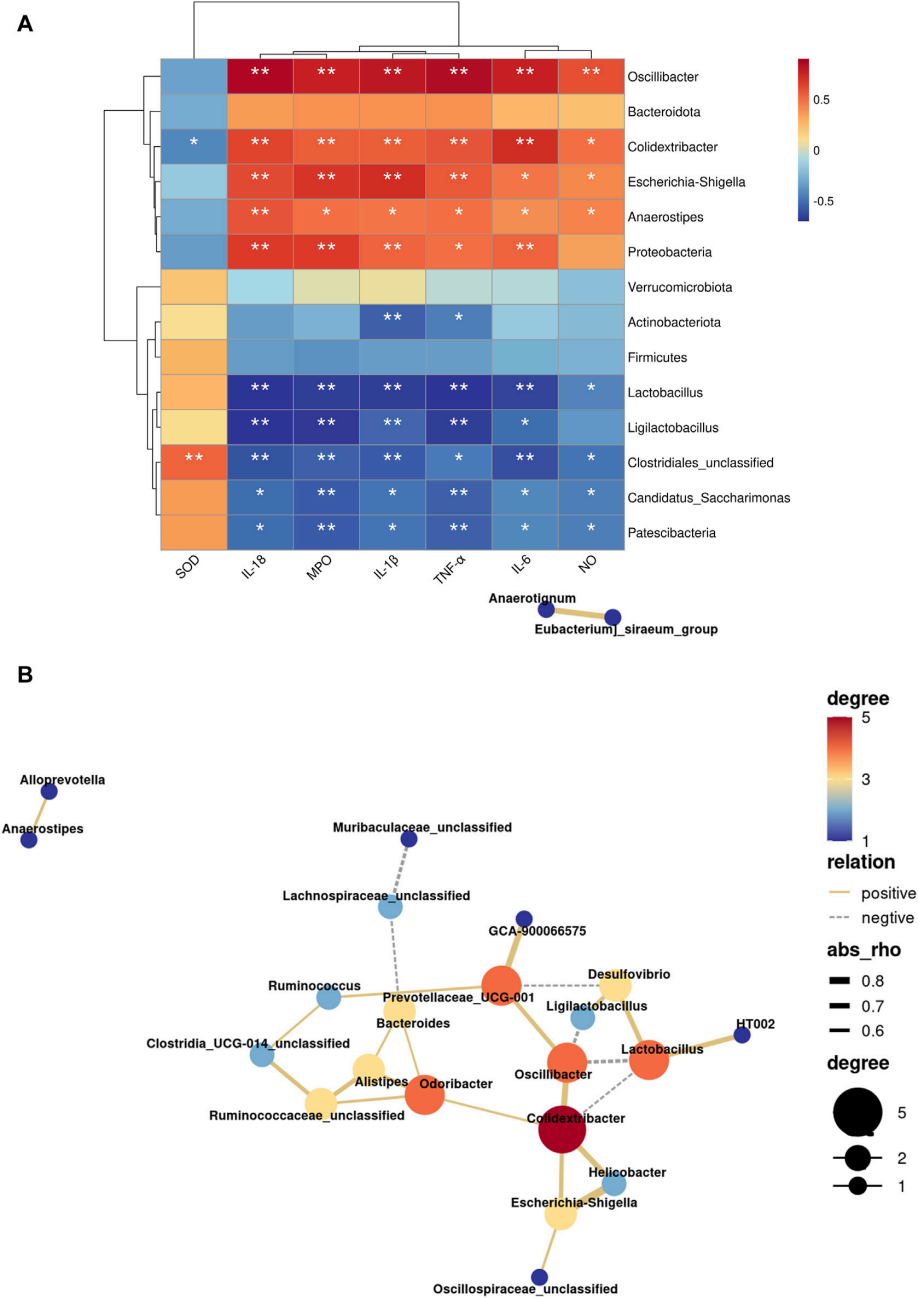
Influence of LHT treatment on the function of the gut microbiota in mice. **(A)** Heatmap of indicator and gut microbiota correlation analysis. **(B)** Network diagram of genus level microbiota correlation analysis. ^*^ *p* < 0.05, ^**^ *p* < 0.01.

Following that, we use the Spearman correlation test to analyze the relationship between several bacteria with significant levels of gut microbiota genus and biological indicators. *Oscillibacter*, *Colidextribacter*, *Escherichia-Shigella*, *Anaerostipes* and Proteobacteria were significantly positively correlated with MPO and inflammatory factor (TNF-α, IL-1β, IL-6, IL-18) (*p* < 0.05, Figure 7A), in contrast to *Lactobacillus*, *Ligilactobacillus*, *Clostridiales_unclassified*, *Candidatus_Saccharimonas* and Patescibacteria. NO was significantly negatively correlated with *Oscillibacter*, *Colidextribacter*, *Escherichia-Shigella*, and *Anaerostipes*, and was significantly positively correlated with *Lactobacillus*, *Clostridiales_unclassified*, *Candidatus_Saccharimonas*, and Patescibacteria (*p* < 0.05). SOD, on the other hand, showed a significant negative correlation with *Colidextribacter* and a significant positive correlation with *Clostridiales_unclassified*.

Meanwhile, we discovered interactions in 21 bacteria genera, with 20 positive and 6 negative correlations (Figure 7B). *Oscillibacter* and *Colidextribacter* were linked in a variety of ways. *Oscillibacter* was positively connected with *Colidextribacter* and negatively correlated with *Ligilactobacillus* and *Lactobacillus*, according to network analysis. Furthermore, *Colidextribacter* was found to be positively connected to *Escherichia-Shigella* and negatively related to *Lactobacillus*. As a result, *Oscillibacter*, *Colidextribacter*, and *Lactobacillus* may be the most important nodal microorganisms.

**FIGURE 8:**
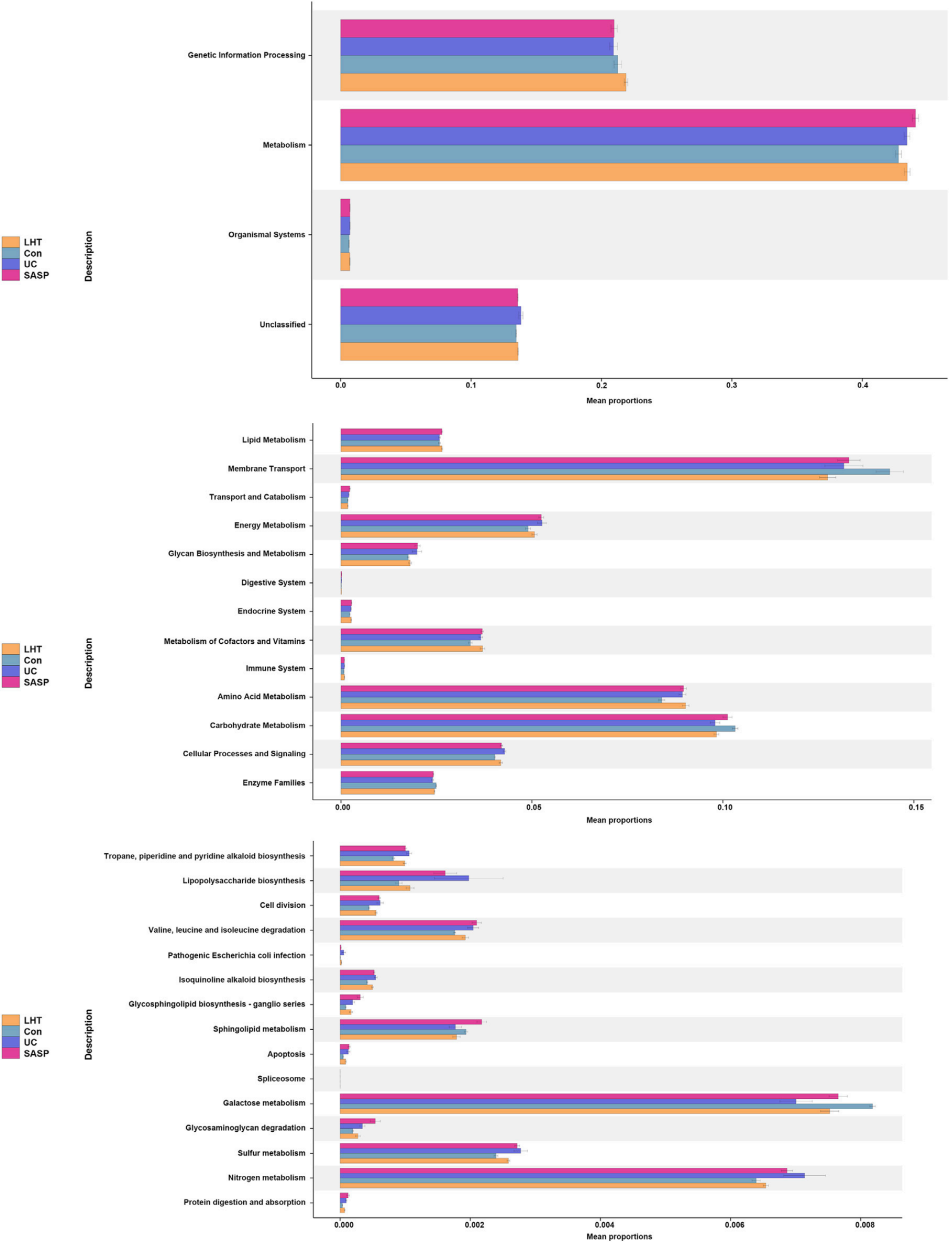
KEGG pathway enrichment analysis.

Meanwhile, the Kyoto Encyclopedia of Genes and Genomes (KEGG) function prediction based on PICRUST2 demonstrated that the mouse gut microbiota was important in metabolic control (Figure 8). The main aggregation sites were galactose metabolism and nitrogen metabolism signaling pathways. Our research revealed that LHT played a significant role in controlling the gut microbiota in the treatment of DSS-induced colitis.

## 4 Discussion

The gut microbiota, a vast and diverse micro-ecosystem, plays a critical role in maintaining a dynamic equilibrium with the host’s organic system. Perturbations in the gut microbiota, also known as ecological dysbiosis, are essential in the onset, progression, and exacerbation of IBD. Disruption of this equilibrium leads to a decline in gut microbiota diversity within the intestinal environment, accompanied by a decrease in beneficial commensal bacteria (Liu et al., 2022). This disruption in the gut environment also leads to the development of abnormal immune responses and a massive accumulation of inflammatory substances, exacerbating and perpetuating the course of IBD (Zhang et al., 2021).

UC is a subtype of IBD. DSS-induced colitis in mice is a widely used animal model for studying IBD. The DSS-induced IBD model mimics human ulcerative colitis by causing increased intestinal epithelial cell permeability in mice. This leads to *in vivo* diarrhea and blood in the stool. Prolonged inflammatory injury further results in colon lengthening and weight loss (Fei and Xu, 2016). In this study, we used 2.5% DSS to induce an IBD mouse model. Our research showed that the mice in the model group exhibited a significant weight loss trend. Meanwhile, the length of the colon was significantly shortened and displayed symptoms of colonic damage. Oxidative stress is closely intertwined with the inflammatory response in the pathogenesis and progression of UC. When cells undergo oxidative stress, the resulting cellular damage promotes and prolongs the inflammatory process (Li et al., 2024). MPO is prevalent in neutrophils and is a prominent candidate for promoting oxidative tissue damage during inflammation (Nadel et al., 2023). Excess NO is a crucial mediator of the inflammatory response triggered by inducible nitric oxide synthase (iNOS) activity (Oppeltz et al., 2012). SOD protects against intestinal inflammation and oxidative stress in IBD (Guan and Lan, 2018). It can effectively improve IBD by destroying reactive oxygen species (ROS) *in vivo* (Shainkin-Kestenbaum et al., 1990) and eliminating the inflammatory response (Zhou et al., 2022). Integrate with our results, the levels of MPO and NO have been increased in model group mice, which indicates that IBD mice have inflammatory damage. SOD levels decreased, reflecting the fact that IBD mice have reduced antioxidant capacity and increased risk of oxidative damage *in vivo*. The NRF2/HO-1 signaling pathway is an important antioxidant pathway that has a significant impact on the progression of IBD (Hwang et al., 2019). As a transcriptional activator, NRF2 can successfully reduce the oxidative stress response by attaching to antioxidant-responsive elements in the promoter regions of target genes (Gomez et al., 2016). Numerous experimental evidence support that the activation of NRF2 not only helps to maintain intestinal integrity but also regulates the expression of inflammatory factors, thus effectively preventing the occurrence and development of UC (Bae et al., 2022; Kim and Jeon, 2022; Peng et al., 2023). Moreover, HO-1, a crucial gene regulated by NRF2, demonstrates noteworthy anti-inflammatory and antioxidant characteristics (Boyanapalli et al., 2014). Increased expression of HO-1 helps to reduce the levels of inflammation-associated cytokines, such as TNF-α, IL-6, IL-1β, and IL-18, which in turn attenuates the inflammatory response (Mills et al., 2018; Xu et al., 2020; Kang et al., 2022; Niu et al., 2024). Our results suggest that LHT plays an important role in IBD therapy by increasing SOD levels, decreasing MPO and NO content, and inhibiting the release of inflammatory factors, such as TNF-α, IL-1β, IL-6, and IL-18, and through the NRF2/HO-1 signaling pathway.

Microbiota plays a crucial role in the inflammatory response in DSS-induced mice. To investigate the effects of LHT on gut microbiota and inflammatory response in DSS-induced mice, we measured the gut microbiota composition of the intestinal contents of mice. DSS consumption reduced gut microbial diversity, as evidenced by decreased α-diversity (Chao1, Shannon, and Simpson indices) and a significant difference in β-diversity. Furthermore, the structural composition of the gut microbiota changed. At the phylum level, mice with colitis had an increase in Bacteroidota and Proteobacteria and a decrease in Firmicutes, Patescibacteria, and Actinobacteriota. At the genus level, the same dissimilar effects occurred. The reduction of the beneficial bacteria *Clostridiales_unclassified*, *Lactobacillus*, *Ligilactobacillus*, *Candidatus_Saccharimonas*, the increase of pathogenic bacteria *Oscillibacter*, *Colidextribacter*, *Anaerostipes*, and *Escherichia-Shigella*, all of which reflect the disturbance of the gut microbiota under the conditions of DSS. As a commonly used first-line drug for the treatment of IBD in the clinic, SASP undoubtedly has a certain degree of efficacy. Furthermore, it has also been used as a frequently utilized positive control drug in mouse experiments aimed at treating IBD (Zhang et al., 2018; Gu et al., 2021). However, concerns about potential safety issues and side effects of current treatments have driven the urgent search for improved medications. TCM has emerged as a promising option due to its reputation for safety and minimal side effects, making it highly valued in clinical practice. Our formula LHT is primarily composed of *Salvia plebeia* R. Br. and *Rhodiola crenulata* (Hook. f. et Thoms.) H. Ohba. These are complemented by auxiliary botanical ingredients including *Lithospermum erythrorhizon* Siebold & Zucc., *Rhus chinensis* Mill., *Pulsatilla chinensis* (Bunge) Regel, *Codonopsis pilosula* (Franch.) Nannf. and *Atractylodes macrocephala* Koidz. These auxiliary herbs traditionally address symptoms by clearing heat and cooling blood, detoxifying and dispersing stagnation, benefiting energy, and moistening the intestines. Notably, treatment with LHT resulted in a reversal of the gut microbiota structure, as evidenced by an increase in the abundance of Firmicutes, Patescibacteria, and Verrucomicrobiota, as well as a decrease in the abundance of Proteobacteria. The therapeutic effect of LHT is comparable to SASP.

*Oscillibacter* has been implicated in IBD, which is capable of producing LPS. It was found that *Oscillibacter* was enriched in DSS mice, which may lead to more severe intestinal inflammation (Li et al., 2018; Wang et al., 2018). Furthermore, it was also associated with intestinal permeability, and mice were shown to have increased *Oscillibacter* abundance after being fed a high-fat diet (Lam et al., 2012). *Oscillibacter* may directly assist in controlling intestinal permeability, or it could be a result of interactions among other microorganisms (Wu et al., 2019). Similar to *Oscillibacter*, *Colidextribacter*, as a typical pathogen, produces inflammatory metabolites that can exacerbate inflammatory responses. Its abundance increased after DSS treatment (Dong et al., 2022). As a probiotic closely related to inflammation and host immunity, *Candidatus_Saccharimonas*, showed a decrease in abundance after DSS treatment (Shao et al., 2021; Wang et al., 2022). Studies suggest the mechanism may involve the regulation of metabolism and energy supply, leading to effective inhibition of inflammatory responses, reduced oxidative stress, and acceleration of the intestinal mucosal repair process (Shao et al., 2021). We explored the pathogenesis of IBD by comparing the correlation between microbiota and IBD inflammatory indicators. It was found that IBD indicators (MPO, NO, TNF-α, IL-1β, IL-6, IL-18) were positively correlated with pathogenic bacteria *Oscillibacter* and *Colidextribacter*, and negatively correlated with beneficial bacteria *Lactobacillus* and *Ligilactobacillus*, *Candidatus Saccharimonas* and Patescibacteria. This further explains that the invasion of pathogenic bacteria in the process of IBD may lead to the excessive release of inflammatory factors and exacerbate the inflammatory response. Therefore, we speculate that LHT may treat IBD by decreasing the abundance of *Oscillibacter* and *Colidextribacter* and increasing the abundance of Patescibacteria, *Candidatus_Saccharimonas*, *Lactobacillus* and *Ligilactobacillus*.

The analysis of KEGG pathway prediction revealed a major focus on metabolism. Among them, there was a significant enrichment in carbon metabolism (galactose metabolism) and energy metabolism (nitrogen metabolism). Numerous studies have shown that galactose metabolism is associated with oxidative stress (Girdhar et al., 2023; Li et al., 2023). D-galactose, a naturally occurring form of sugar, can be metabolized in small quantities by conversion to glucose. In contrast, excessive d-galactose accumulation undergoes oxidation by galactose oxidase, generating H_2_O_2_ and a substantial amount of reactive oxygen species (ROS). ROS disrupts intestinal homeostasis through oxidative stress, further exacerbating inflammatory responses (Li et al., 2016). Nitrogen metabolism occupies an important factor in the gastrointestinal tract. There are various sources of nitrogen, including inorganic and organic nitrogen. Intestinal epithelial cells produce iNOS, which can be converted to NO with L-arginine as a substrate with reactive oxygen and nitrogen species (Sowers et al., 2022). Ammonia, a major byproduct of nitrogen metabolism, is sourced in large quantities from the small bowel and colon (Campion et al., 2019). Microorganisms respond to exogenous environmental stresses in the gut by utilizing metabolism substances. Proteobacteria has been observed to exhibit enrichment in genes associated with nitrogen utilization (Ni et al., 2017), and *Escherichia coli* is extensively recognized for its involvement in nitrogen metabolism (Brown et al., 2014). They can synthesize amino acids using nitrogen sources in the gut and participate in nitrogen metabolism (Yin et al., 2022). It is well established that *Lactobacilli* has the ability to metabolize galactose (Jung et al., 2016). Our results suggested that LHT may alleviate the inflammatory response by attenuating the level of oxidative stress by a mechanism that may be related to the regulation of gut microbiota. These discoveries not only yield fresh insights into the pathophysiological mechanisms underlying IBD but also furnish substantial support for the advancement of more efficacious therapeutic modalities.

## 5 Conclusion

**FIGURE 9:**
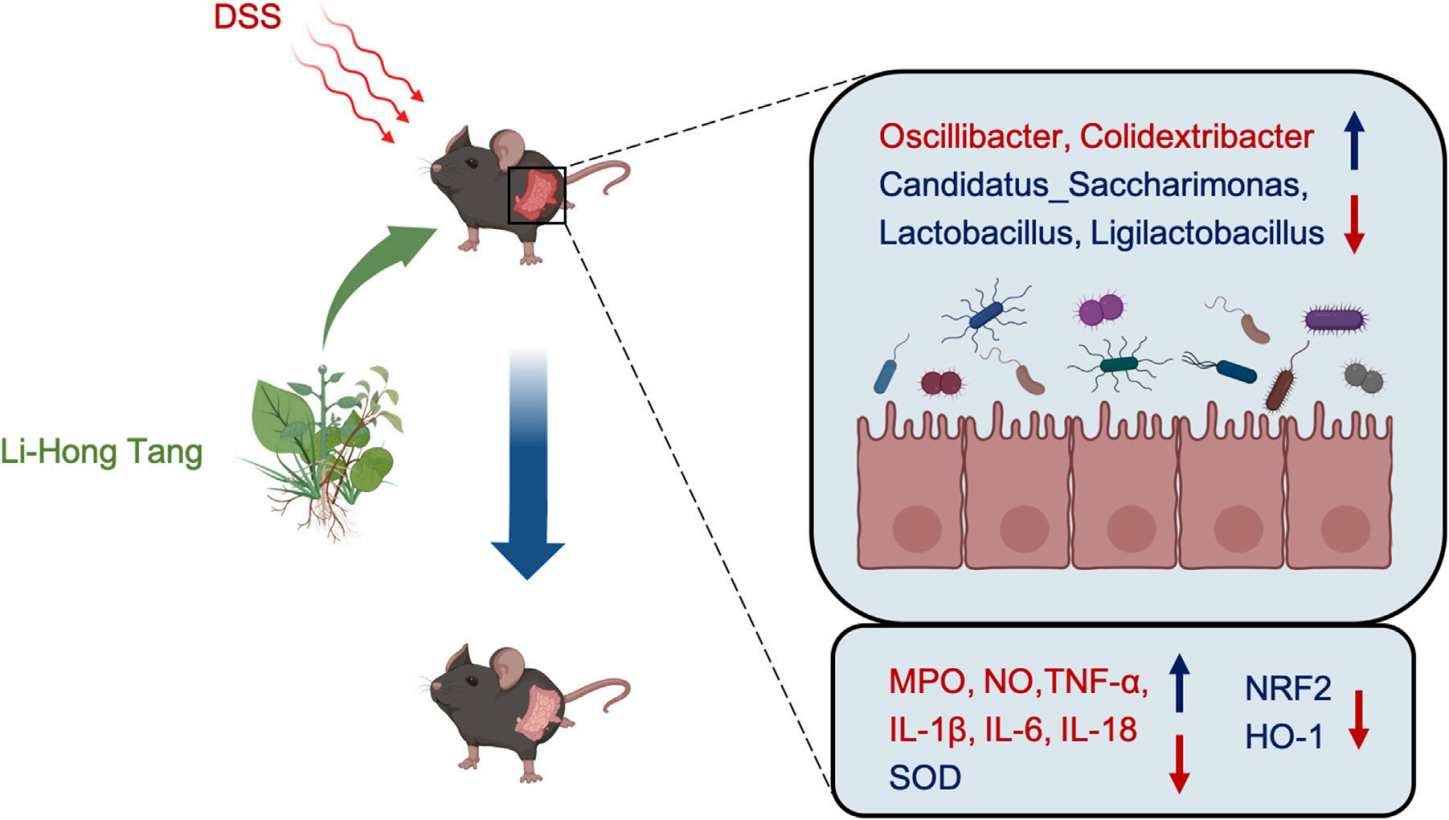
The mechanism of action of LHT on UC is depicted in the diagram. LHT alleviates UC by modulating gut microbiota and signaling pathways to reduce UC-induced oxidative stress and inflammation.

Our study demonstrated that LHT intervention in colitis mice effectively reduced inflammatory markers and oxidative stress, leading to the amelioration of colitis symptoms. This therapeutic effect appears to be mediated through the modulation of gut microbial composition and the NRF2/HO-1 signaling pathway. Figure 9 illustrates the mechanism underlying the impact of LHT in DSS colitis mice. These findings suggest that LHT, which is composed of Chinese herbal medicine, promises to be a therapeutic alternative for colitis treatment. However, further investigation of its efficacy and safety in clinical trials is needed.

## Data Availability

The datasets presented in this study are deposited in the Figshare, the link is dx.doi.org/10.6084/m9.figshare.25808563.

## Abbreviations

UC, Ulcerative Colitis; IBD, Intestinal Inflammatory Disease; TCM, Traditional Chinese Medicine; LHT, Li-Hong Tang; DSS, Dextran Sulfate Sodium; SASP, Sulfasalazine; MPO, Myeloperoxidase; NO, Nitric Oxide; SOD, Superoxide Dismutase; DAI, Disease Activity Index; MS, Mass Spectrometer; KEGG, Kyoto Encyclopedia of Genes and Genomes; iNOS, inducible Nitric Oxide Synthase; ROS, Reactive Oxygen Species.
